# Factors associated with motivation in medical education: a path analysis

**DOI:** 10.1186/s12909-018-1256-5

**Published:** 2018-06-18

**Authors:** Natchaya Kunanitthaworn, Tinakon Wongpakaran, Nahathai Wongpakaran, Salilthip Paiboonsithiwong, Natchaphon Songtrijuck, Pimolpun Kuntawong, Danny Wedding

**Affiliations:** 1 Faculty of Medicine, Chiang Mai University, Chiang Mai, Thailand; 2 Department of Psychiatry, Faculty of Medicine, Chiang Mai University, 110 Intawaroros Rd., T. Sriphum, A. Muang, Chiang Mai, 50200 Thailand; 3 Saybrook University, Oakland, CA USA

**Keywords:** Medical education, Mental health, Motivation, Personality trait, Social support, Medical students

## Abstract

**Background:**

This study identified and investigated the relationship between demographics, mental health problems, positive personality traits and perceived social support and motivation in medical education (MME) among first year medical students.

**Methods:**

One hundred-thirty eight first year medical students completed the Academic Motivation Scale, Outcome Inventory, Strength Based Inventory, and Multidimensional Scale for Perceived Social Support. Path analysis was conducted to identify relationships between the variables of interest and each type of motivation, including intrinsic and extrinsic motivation and amotivation.

**Results:**

The mean age of the sample was 18.86 ± 0.74 and 60% of the subjects were female. Path analysis showed that extrinsic motivation was positively associated with being female, personal choice for studying medicine, and grade point average at high school. Intrinsic motivation was correlated with perceived family support, personal choice for studying medicine and the positive attribute of determination. Amotivation was related to being male, personal choice, and depression. While both extrinsic and intrinsic motivation were correlated, they were uncorrelated with amotivation. All variables accounted for 18, 13, and 45% of variance of intrinsic motivation, extrinsic motivation and amotivation, respectively.

**Conclusion:**

Each type of motivation has different but related predictors. Extrinsic and intrinsic motivation can be promoted, whereas amotivation represents an exclusive issue, one related more to depression, that needs to be reduced to not interfere with academic achievement and quality of life of medical students.

## Background

Many factors influence the decision to become a doctor. One study revealed that students chose this profession because of the humanistic aspects of medicine, openness to new experiences, a deep personal identification with the profession, a critical need for fulfillment in their careers and because of their desire to help people and be recognized for their usefulness [1]. In studying medicine, a high level of motivation is required for learning [2], academic success and the intention to continue studying medicine [3], and the development of professional identity [4]. In addition, academic achievement can influence and affect motivation for medical education [3]. According to Deci and Ryan’s Self-determation Theory, there are two types of motivation, intrinsic and extrinsic. Motivation can be more or less autonomous, and it can take the form of intrinsic or extrinsic amotivation. Intrinsic motivation reflects the human propensity to learn and assimilate, while extrinsic motivation results from either external control or true self-regulation [5]. Lack of motivation is typically catagorized as no motivation or amotivation [6].

Extrinsic motivation can be ranked along a continuum; it includes external regulation (reward/ punishment), introjected regulation (self-control, internal reward or punishment), identified regulation (personal importance, conscious valuing), and integrated regulation. Intrinsic motivation involves interest, enjoyment, and inherent satisfaction [7]. Another common way for investigators to describe motivation is autonomous vs. controlled motivation; autonomous motivation is a combination of identified regulation, integrated regulation, and intrinsic motivation, whereas a combination of external regulation and introjected regulation defines controlled motivation [8].

Motivation in medical education can be influenced by multiple factors. Kusurkar reviwed and described (1) manipulatable variables, such as autonomy, competency, and relatedness, and (2) unmanipulatable variables such as age, gender, and ethnicity, all of which can influence motivation. Likewise, Orsini and colleagues conducted a systemic review of factors influencing motivation and categorized them into five groups: 1) intrapersonal determinants such as age and gender; 2) interpersonal determinants such as academic conditions; 3) cognitive outcomes such as beliefs; 4) affective outcomes such as anxiety or depression; and 5) behavioral outcomes such as academic engagement [9]. Parental support and encouragement had a positive effect on motivation, whereas lack of teacher support had a negative effect [10]. Choice, acknowledgment of feelings, and opportunity for self-direction were also found to enhance intrinsic motivation because these factors facilitated a greater sense of autonomy [11].

Personality traits and positive attibutes were found to be significantly correlated with motivation, e.g. love of learning, perseverance, and gratitude. The strongest correlations with positive classroom behavior were found for perseverance, self-regulation, prudence, social intelligence, and hope [12]. On the other hand, some personality traits and strengths were also found to be positively related to depression [13].

Motivation can also be influenced by geographical and economic differences. A recent literature review summarized that motivation for security in work and finance was found in lower-income countries while motivation for humanitarian purposes was found in the high-income countries. However, it is interesting to note that despite the fact that countries in Asia differ significantly in per capita wealth, they still share a collectivistic familial background (e.g. Singapore, India, and Iran). One study found that this family background influenced medical students’ motivation [14]. This family influence is also found in cultures outside Asia [15].

Like most Asian countries, Thailand is a collectivistic society. The motivation for studying medicine is linked to family support in addition to students’ personal desires. Most students enter medical school directly from high school; as a result, parental influence is inevitable. Medicine is always a top career choice for highly academically achieved high school students, and the motivation to pursue a medical education often comes from parents rather than the students themselves. Low motivation for studying has been shown to result in high levels of anxiety and depression [13, 16, 17].

Based on the previous findings and our own cultural background, we sought to identify the relationship between motivation and related variables including 1) individual factors (i.e. gender, personal preferences for medicine, positive attributes and traits, and depression), 2) an academic achievement factor (i.e. grade point average at high school), and 3) a family factor (i.e. support for studying medicine).

We were interested in testing these variables within our cultural context, knowing that the factors associated with motivation may vary widely from those found in other cultures. In addition, we were specifically interested in motivation to study medicine (for freshmen) rather than motivation to continue studying medicine (in the later years) because understanding these factors might make early detection and intervention possible for those students who have low motivation or amotivation. To the best of our knowledge, these potential predictors has not been studied together in the first year medical students.

We hypothesized, based on Ryan and Deci’s hierarchy of motivation, that intrinsic motivation would be related to personal preference for medicine and positive personality traits, while extrinsic motivation and amotivation would be related to gender, grade point average, depression, and family support.

**Fig. 1:**
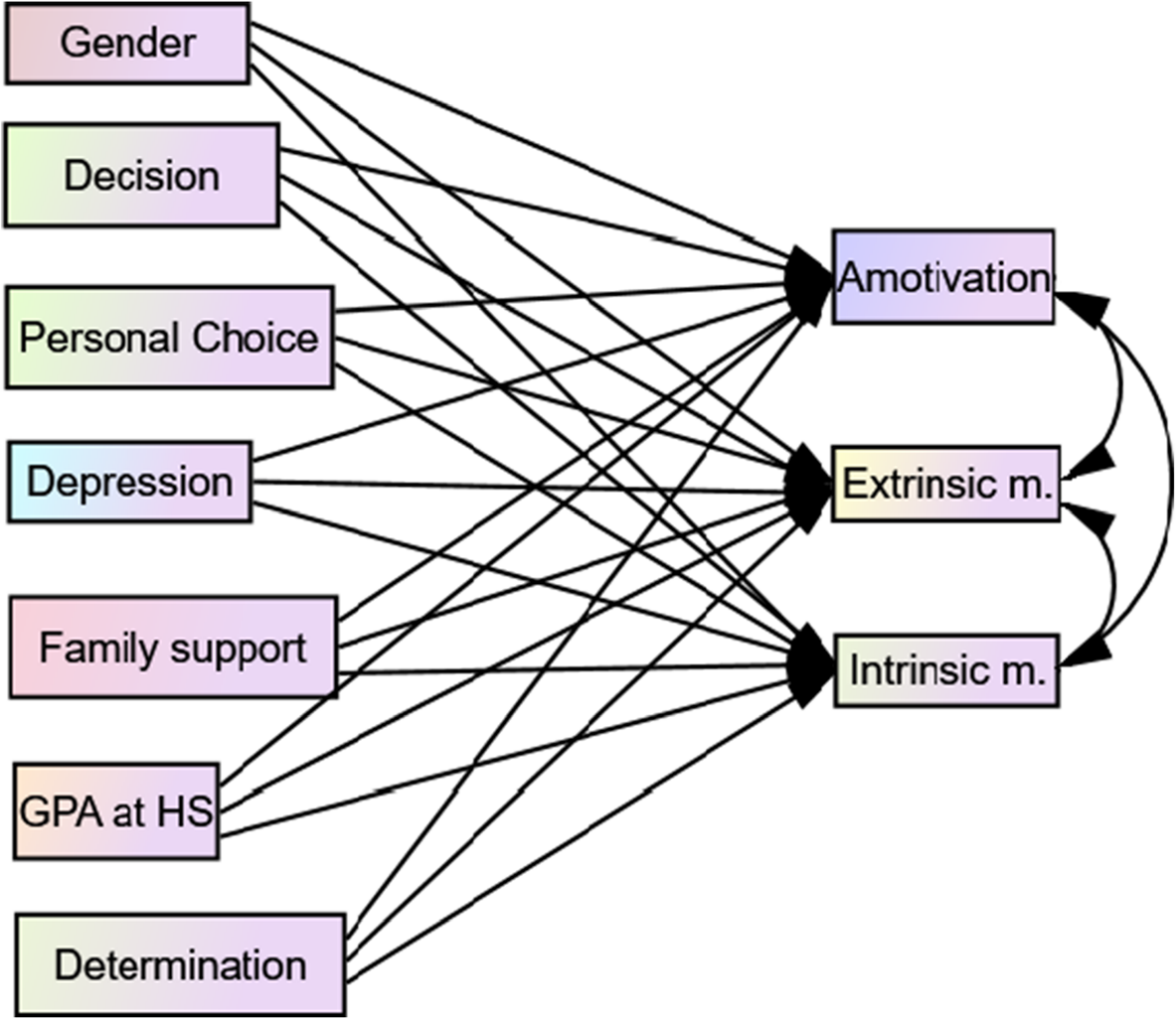
The proposed hypothesized model model illustrating causal paths linking variables with motivation. GPA in HS = Grade Point Average in high school; Extrinsic m. = Extrinsic motivation; Intrinsic m. = Intrinsic motivation. The lines with arrowheads show the direction of the path coefficients

The model was tested using path analysis within a structural equation model framework (Fig. 1).

The following main hypotheses were tested: First, consistent with previous research, it was expected that male would be positively associated with amotivation, whereas female would be positively associated with intrinsic motivation or extrinsic motivation. Personal choice (preference) for medicine was expected to be positively associated with both intrinsic and extrinsic motivation, but negatively associated with amotivation. In addition, it was hypothesized that both positive personality traits and perceiving family support would be associated with intrinsic or extrinsic motivation. Finally, it was predicted that the high school GPA and depression would be associated with extrinsic motivation.

## Methods

### Study design

This research involved an observational study of first-year medical students at a university in northern Thailand who began their medical education in Academic Year 2014.

### Participants

A total of 140 of the 250 first-year medical students attending Chiang Mai University were recruited in the 2014 academic year. These students were asked to provide demographic data including completing questionnaires related to decisions and preferences regarding medical education. The 4 measures assessed motivation, mental health problems, perceived social support and positive personal traits or inner strength.

### Measurements

#### Academic motivation scale (AMS)

The AMS, developed by Vallerand et al., and adapted for use among medical students by Kusurkar et al., measures three types of motivation based on the self-determination theory (SDT), i.e., intrinsic, extrinsic and amotivation [18, 19]. The AMS has 28 items that measure motivation for studying medicine, using a 7-point Likert scale that ranges from 1, “strongly disagree,” to 7, “strongly disagree.” The three subscales of intrinsic motivation included 1) knowledge gained, 2) accomplishment and 3) stimulation. Extrinsic motivation also had three subscales: 1) identified regulation, 2) introjected regulation and 3) external regulation. The Thai version was developed by Tinakon Wongpakaran (unpublished data, 2015) and was used with permission. The internal consistency of the Thai version of the AMS was excellent (Cronbach α = 0.84 for all items, 0.91 for amotivation, 0.83 for intrinsic motivation, and 0.85 for extrinsic motivation).

#### Outcome inventory (OI-21)

The Outcome Inventory [20] is a self-rating questionnaire that measures four common mental health problems: anxiety, depression, interpersonal problems, and somatic complaints. It includes 21 questions assessed with Likert scales that range from 1 (not at all) to 5 (very much). A higher score indicates a higher level of psychopathology. The Cronbach α value (0.92) was excellent.

#### Multidimensional scale for perceived social support (MSPSS)

The MSPSS [21] measures perceived social support from three sources: friends, family, and significant others. It contains 12 items with Likert scales that range between 1 (very strongly disagree) and 7 (very strongly agree). A higher score indicates a higher level of feeling supported. The Thai version [22] has a Cronbach’s α of 0.90 with good concurrent validity. The confirmatory fit index was 0.95, and the root mean square error of approximation was 0.055.

#### Strength-based inventory (SBI)

The SBI was developed by Wongpakaran & Wongpakaran [23]. It is a ten-item multiple-choice scale assessing ten positive personality traits (strengths): generosity, perseverance, truthfulness, loving-kindness, wisdom, determination, morality/virtue/precept, patience, equanimity and mindfulness. A higher score indicates a higher level of a given trait. Using Rasch analysis, the SBI showed satisfactory construct validity; the internal consistency was also acceptable (Cronbach’s alpha = 0.72; person separation reliability = 0.72; item separation reliability = 0.99).

### Statistical analysis

Descriptive statistics were used to assess demographic data such as gender and the decision to study medicine. Correlations among variables were analyzed using Pearson’s correlation for continuous variables, and point-biserial correlation for dichotomous-continuous variables. All demographic data, mental health problems and psychosocial scores were analyzed to identify meaningful correlations. Significant variables were included in the path model. Two cases that were outliers were deleted. We then performed path analyses to investigate the effect of relevant independent variables on intrinsic motivation, extrinsic motivation, and amotiovation. The regression/path coefficients were all in standardized form (β). The bootstrapping method was used since the assumption of a normal multivariate distribution was not met. Model fit was also assessed using the following criteria: a chi-square/df of ≤2, a *P*-value of > 0.05, a comparative fit index of ≥0.95, Tucker-Lewis Index ≥0.95 and a root mean square error approximation of < 0.06 [24]. The data were analyzed using IBM SPSS for Windows, version 22.0 (IBM Company, Armonk, NY, USA); AMOS version 18 and Mplus version 8 were used for path analysis.

### Ethical approval

This study was approved by the Ethics Committee of the Faculty of Medicine, Chiang Mai University, Thailand, (IRB no. 004/2015). Informed consent forms from the participants were obtained.

## Results

The study included 138 first-year medical students. Sixty percent were female with a mean age of 18.86 ± 0.74 years. Most students independently chose medicine as a career, most often with parental agreement. Other details including types of motivation and details of each subscale are described in Table 1.

**Table 1:** Descriptive data at baseline and 6-month follow-up (*N* = 138)

Variables	*N* (%)
Sex	
Female	84 (60)
Male	76 (40)
Age (years): Mean (SD)	18.86 (0.74)
Family income (USD/Year)	
<14 k	22.6
14 k–21 k	27.8
21.1 k–28 k	12.8
28.1 k- 35 k	15
>35.1 k	21.8
Father’s years of education: Mean (SD)	14.13 (6.4)
Mother’s years of education: Mean (SD)	12.93 (6.2)
Decision to study medicine (*N* = 137)	
Oneself	112 (81.9)
Others (parents, peers, teachers)	25 (18.1)
Medicine is a personal choice (*N* = 136)	
Yes	88 (64.2)
No	48 (35.8)
Parental agreement (*N* = 138)	
Yes	123 (88.5)
No	15 (11.5)
Clinical characteristics: Mean (SD)	
Motivation	
Total score	137.86 (20.3)
Intrinsic motivation	55.96 (9.0)
Obtaining knowledge	20.45 (3.36)
Accomplishment	18.34 (4.55)
Stimulation	17.15 (3.70)
Extrinsic motivation	60.47 (11.6)
Identified regulation	21.20 (3.64)
External regulation	20.67 (4.29)
Introjected regulation	19.03 (7.0)
Amotivation	10.56 (5.3)
Outcome Inventory	
Total score	40.77 (10.7)
Anxiety	14.17 (4.2)
Depression	7.63 (2.7)
Interpersonal problems	8.16 (3.1)
Somatization	10.82 (3.5)
MSPSS	
Total score	66.31 (10.6)
Significant others	19.38 (6.1)
Family	24.78 (3.4)
Friends	21.18 (4.2)
Positive personality trait (strength)	
Total score	38.68 (7.5)
Truthfulness	3.31 (1.3)
Perseverance	3.60 (1.2)
Wisdom	3.29 (0.8)
Generosity	4.06 (1.4)
Morality/Virtue/Precept	3.91 (1.7)
Meditation, mindfulness	2.34 (1.1)
Patience	4.47 (1.4)
Equanimity	4.46 (1.1)
Determination	4.55 (1.8)
Loving-kindness	3.88 (1.5)
^a^GPA in high school: Mean (SD), min -max	3.91 (0.1), 3.25–4.00

*SD* standard deviation

^a^Grade Point Average in high school

### MME and its correlated variables

Significant correlations were found for sex, decision maker and all types of motivation, except for intrinsic. Personal choice for medicine was significantly correlated with total motivation and all types of motivation. In contrast, parental agreement was not correlated with any other variables. The same was true for parents’ education (Table 2).

**Table 2:** Correlation of demographic and psychosocial variables with motivation (*n* = 138)

	Intrinsic motivation	Extrinsic motivation	Amotivation
Age	−.128	−.075	−.005
Sex	.146	.274^a^	−.273^a^
Father’s year of education	−.099	−.102	.076
Mother’s year of education	−.089	−.083	.012
GPA at high school	.240^a^	.329^a^	−.302^a^
Decision maker	−.187^b^	−.225^a^	.274^a^
Family agreement	−.038	−.037	.087
Personal choice	.369^a^	.235^a^	−.549^a^
MSPSS			
Significant others	.281^a^	.060	−.191^b^
Family	.289^a^	.197^b^	−.249^a^
Friends	.283^a^	.077	−.189^b^
SBI			
Truthfulness	−.023	−.073	−.096
Perseverance	.264^a^	.183^b^	−.146
Wisdom	.179^b^	−.088	−.133
Generosity	.037	−.025	−.072
Virtue, Precept	.069	.047	−.112
Meditation, mindfulness	.154	.057	−.191^b^
Patience	.193^b^	.206^b^	−.088
Equanimity	.203^b^	.097	−.078
Determination	.316^a^	.189^b^	−.237^a^
Loving-kindness	.132	.134	−.167^b^
OI			
Anxiety	−.188^b^	−.004	.320^a^
Depression	−.252^a^	−.085	.539^a^
Interpersonal problems	−.217^b^	−.016	.239^a^
Somatization	.042	.197^b^	.142

*MSPSS* Multidimensional Scale of Perceived Social Support, *SBI* Strength-Based Inventory, *OI* Outcome Inventory

^a^ Correlation is significant at the 0.01 level (2-tailed)

^b^ Correlation is significant at the 0.05 level (2-tailed)

Results showed that there was no correlation between high school GPA and intrinsic and extrinsic motivation, but high school GPA was significantly and negatively correlated with amotivation (*r* = − 0.362, *P* < 0.01) and positive correlated with extrinsic motivation (*r* = .226, *p* < .01). Intrinsic motivation and amotivation were significantly correlated with depression, anxiety, and interpersonal problems, while extrinsic motivation had significant correlation only with somatization. The same was true for perceived social supports in that these variables correlated more with intrinsic motivation and amotivation. Perceived family support was found to have the strongest relationship with all types of motivation. Among motivation subscales, intrinsic motivation was positively correlated with extrinsic motivation (*r* = .559, *p* < .001), but negatively correlated with amotivation (*r* = −.349, p < .001). Extrinsic motivation also had negative correlation with amotivation (*r* = −.180, *p* = .035).

For SBI items, intrinsic motivation correlated with perseverance, wisdom, patience, equanimity, meditation/mindfulness, determination, and loving-kindness. Perseverance and determination exhibited the strongest relationship among all SBI items.

**Fig. 2:**
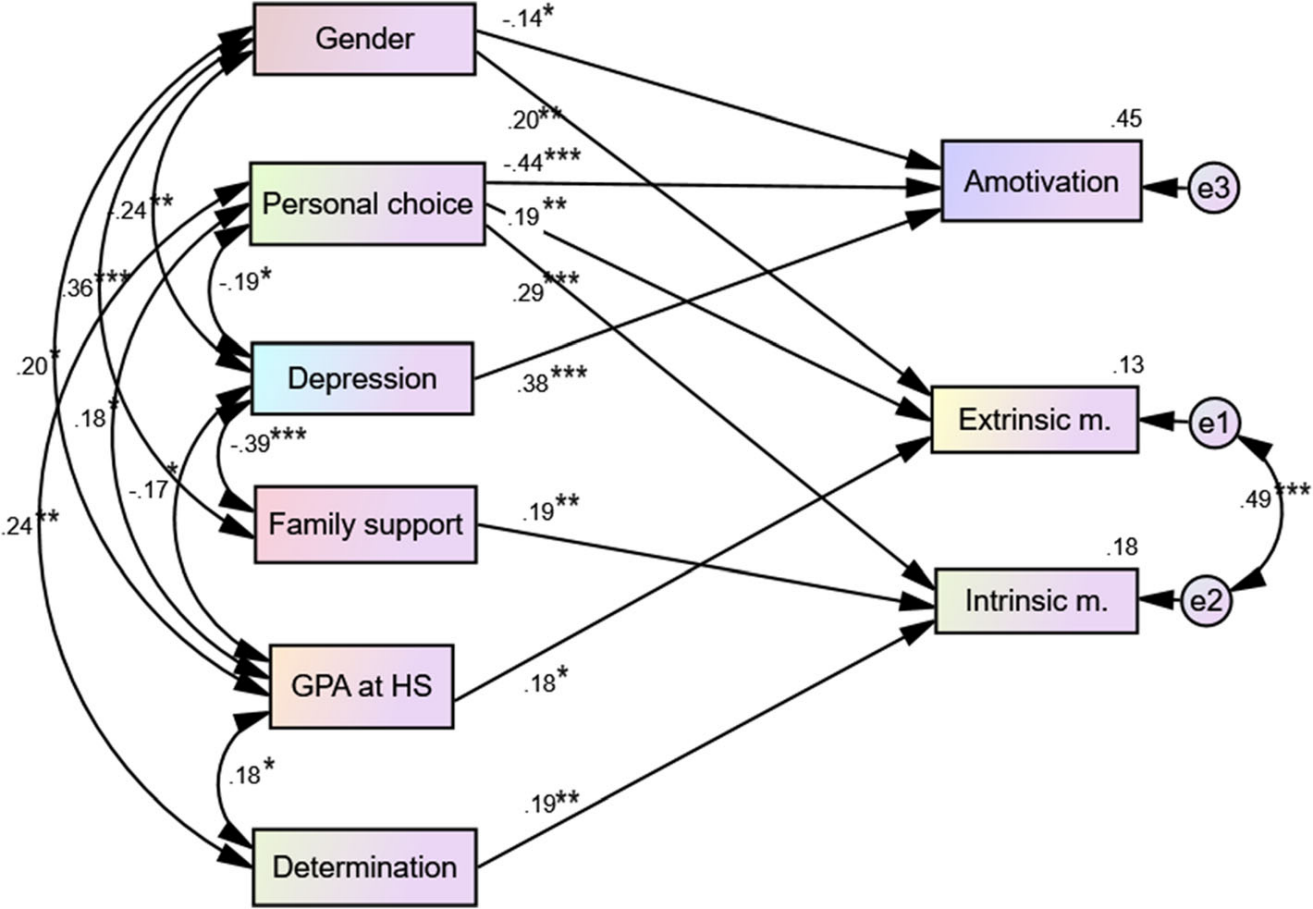
The final path model illustrating direct and indirect effects and causal paths linking variables with motivation. GPA in HS = Grade Point Average in high school; Extrinsic m. = Extrinsic motivation; Intrinsic m. = Intrinsic motivation. Values on the lines = path coefficient or standardized coefficient; *P < 0.05, **P < .01, ***P < .001, NS = non-significant. Values on the lines were standardized regression coefficients. Value on Amotivation, Extrinsic m., and Intrinsic m. in the rectangle are percent variance explained by each variable (R^2^). The model fit statistics were as follows: chi-square = 24.460, *df* = 17, chi-square/*df* = 1.439, *P* = 0.107; comparative fit index =0.971, Tucker-Lewis Index = 0.939, and root mean square error approximation =0.057 (90%CI = 0.000–0.103)

Path analysis results showed that depression, and personal choice had stronger relationships to amotivation than high school GPA (standardized regression coefficient (β) = 0.38 (*t* = 5.785) for depression, − 0.44 (*t* = − 6.804) for personal choice, and − 0.14 (*t* = − 2.189) for gender). Gender and high school GPA had significant relationship to extrinsic motivation (β = 0.20 (*t* = 2.719) for gender and β =0.18 (*t* = 2.539) for high school GPA). Personal choice, perceived family support and determination had direct effect on intrinsic motivation with β 0.29 (*t* = 3.707), 0.19 (*t* = 2.739), and 0.19(*t* = 2.698) respectively. Also, a significant relationship was found between extrinsic motivation and intrinsic motivation (*r* = 0.49, *p* < .001), but not with amotivation. This final path model yielded better-fit statistics to the data (Fig. 2). All variables accounted for 18, 13, and 45% of total variance of intrinsic motivation, extrinsic motivation, and amotivation, respectively.

## Discussion

The aim of the present study was to explore the variables related to motivation among first year medical students. The results help us understand motivation in medical education and provide new findings for further research.

In general, the variables studied related to each type of motivation were supported by related research. As expected, personal choice for medicine played an important role in motivation. High school GPA was related to extrinsic motivation, and students with good high school GPAs were highly motivated to study medicine, as medicine is a popular career for high academic achieving students in Thailand. A previous study documented that intrinsic motivation is related to identification with academic variables (e.g. relating to an academic environment and valuing academic achievement) and meaningful cognitive engagement (the significant amount and type of strategies the learners employ) [25]. This finding suggests a good high school GPA is not necessarily the result of high intrinsic motivation. Though no evidence indicates that high grades in high school science courses predict success as a physician, minimum grades when applying for this major are required by almost all medical schools. A history of high grades may not guarantee a students’ academic achievement in medical school because this extrinsic motivation may subside when students actually are in medical school. This is especially true in advanced years of education when other kinds of motivation (rather than only extrinsic motivation) are required.

Gender is an important and relevant predictor. Male medical students showed lower extrinsic motivation, but higher amotivation than females. This was consistent with Kusurkar et al.’s study demonstrating that females showed higher controlled (external + introjected) motivation [6]. The findings were similar to other studies, despite the fact that the motivation scale used in those studies differed from the one used in the current study [26, 27]. The role gender plays in motivation (particularly intrinsic motivation) may be postulated by the path model, which shows that gender is positively correlated with family support and that females tend to be closer to other family members than males [28]. This has been observed to be similar across cultures [29, 30]. This study supported the importance of the family factor – i.e., spending time with family was positively associated with intrinsic motivation. Likewise, Roth et al. found parents’ positive regard linked to autonomy support [31]. Our sample demonstrated the importance of family support, and this variable contributed the most to motivation (affecting intrinsic more than extrinsic motivation). Interestingly, friends (peer groups) had less impact than family among these first year students, emphasizing the strong relationship between the students and their families. Finding a family influence on students’ motivation in medical education was consistent with previous findings [32, 33], and it is clear that families can promote both extrinsic and instrinsic motivation in students. In the present study, family support was most related to intrinsic motivation. This could be contributed to the measure used to assess family support, which did not address specific family encouragement to study medicine, but instead assessed general feelings of perceived support from family. This kind of support, as part of general psychological development, may influence how students develop general positive attributes, motivation, and achievement [34–36], and even their ability to overcome psychological problems [37]. From a psychological point of view, it is important to recognize that support from family is important throughout the life cycle. This has implications for teachers, curriculum developers, and administers who have to design interventions and activities that balance promoting positive support with student autotonomy.

The fact that positive personality traits are related to intrinsic motivation has been documented by related research. Recent findings have demonstrated that intrinsic motivation is positively associated with well-being, meaning in life, and positive emotions, but negatively associated with negative emotions [38]. Orsini et al. found similar results with positive affect and intrinsic motivation [9]. Tanaka et al. found similar results to ours in that persistence was positively associated with intrinsic motivation [30]. Although different measures were used in these two studies, researchers agree that these attributes are related to intrinsic motivation. Interestingly, amotivation was negatively correlated with determination and loving-kindness. This makes sense for determination, and these data are consistent with Cloninger’s findings [39].

This study revealed that positive personal traits, as measured by the SBI, were correlated with intrinsic motivation, especially perseverance and determination. Even though only determination remained in the path model, it did not mean that other traits were of no value. For example, wisdom, equanimity or loving-kindness may be important predictors of well-being. Further study with other traits is encouraged.

In contrast to these findings, amotivation showed a reverse pattern and was more closely related to psychological problems than to real motivation. A relationship between depressive symptoms and amotivation was found in a related study [40]. Lack of motivation can result from depression; likewise, lack of motivation also can lead to depression. We cannot conclude a cause-effect relatiohship from this crossectional study. However, it is clearly useful for teachers to be alert to detect amotivation in students, and they should evaluate students for depression as early as possible.

### Limitations

The data from a small population in one medical school will not be representative or accurately reflect all medical students. First year students’ responses may differ from senior students. The evaluation of academic achievement was limited because only high school GPA or course grades were used, which might not truly reflect students’ academic ability. Moreover, extracurricular activities, educators’ teaching methods, other students’ characteristics or attributes and other relevant factors, which may have affected their motivation, were excluded from the analysis. We used the less stringent approach of path analysis instead of a different measurement model (SEM) because we first wanted to evaluate our hypothesis with a small sample.

### Strength and future research

This study investigated predictors of motivation among medical students using multiple variables (i.e. demographics, mental health problems, positive personality traits and perceived social support). It adds to a large volume of research linking motivation with other predictor variables. The causal relationships among variables, especially between kinds of motivation, need to be studied in future research; in addition, a longterm, longitudinal study will provide more robust evidence regarding cause and effect.

## Conclusions

Females were more motivated than males to pursue a medical education. Personal preference for studying medicine was important and played a vital role in motivation. Gender, family and personal choice were related to each other and important for motivation. Extrinsic motivation and intrinsic motivation were closely interwoven and could be bolstered by a variety of methods; in contrast, amotivation produced a hurdle to medical education, and was related to depression. It is important to monitor medical students’ motivation along with mental health problems, including their perceived family support, which is especially important for first year medical students. Continuing family support should be nurtured and encouraged.

## Abbreviations

AMS: Academic Motivation Scale
GPA: Grade Point Average
MME: Motivation in Medical Education
MSPSS: Multidimensional Scale for Perceived Social Support
OI-21: Outcome Inventory
SBI: Strength-Based Inventory
SDT: Self-Determination Theory
